# Supplementary material for: Compartmentalised RNA catalysis in membrane-free coacervate protocells
Source: Nat Commun. 2018 Sep 7;9:3643. doi: 10.1038/s41467-018-06072-w (PMC6128941; doi:10.1038/s41467-018-06072-w)
Supplement: Supplementary file 1 — Supplementary Information [file 41467_2018_6072_MOESM1_ESM.pdf]

Supplementary Information:

Compartmentalised RNA catalysis in membrane – free coacervate protocells

Björn Drobot[‡], Juan M. Iglesias-Artola[‡], Kristian Le Vay[†], Viktoria Mayr[†], Mrityunjoy Kar[‡], Moritz Kreysing[‡*], Hannes Mutschler[†*], T-Y. Dora Tang[‡*]

‡Max-Planck Institute for Molecular Cell Biology and Genetics, Pfotenhauerstraße 108, 01307, Dresden, Germany

†Max-Planck Institute for Biochemistry, Am Klopferspitz 18, 82152 Martinsried, Germany.

These authors contributed equally: Juan M. Iglesias-Artola, Kristian Le Vay

Content:

Supplementary Table 1-3

Supplementary Figure 1-14

Name	Sequence 5' -> 3'
5T7	GATCGATCTCGCCCGCGAAATTAATACGACTCACTATA
sTRSV_min_wt_TX	TGTGCCTTTCGTCCTCACGGACTCATCAGTTCAGCTCCCTATAGTGAGTCGTATTAATTTC
sTRSV_min_mut_TX	TGTGCCTTTTGTCCTCACGGACTCATTAGTTCAGCTCCCTATAGTGAGTCGTATTAATTTC

Supplementary Table 1: Sequences of DNA oligonucleotides. Inactivating point mutations are highlighted in red

**Name**	**5' tag**	**Sequence 5' -> 3'**	**3' tag**	**Length (nt)**	**M_w (kDa)**	**λ_{exc} (nm)**	**λ_{em} (nm)**
HH-min		GGGAGCUGAACUGAUGAGUCCGUGAGGACGAAAGG CACA		39	12.8		
HH-mut		GGGAGCUGAACUAAUGAGUCCGUGAGGACAAAAGG CACA		39	12.8		
TAM-HH-min		GGGAGCUGAACUGAUGAGUCCGUGAGGACGAAAGG CACA	TAM	39	13.4	544	576
FAM-substrate	FAM	UGCCUCUUCAGC		12	4.3	495	520
FRET-substrate	FAM	UGCCUCUUCAGC	BHQ1	12	4.8	495	520
FAM-cleaved substrate	FAM	UGCCUC		6	2.3	495	520
FAM-Flex	FAM	GCAGAACGAAGC		12	4.6	495	520
FAM-Tet	FAM	GCACUUCGGUGC		12	4.5	495	520

Supplementary Table 2: RNA oligonucleotides. Inactivating point mutations are highlighted in red

	k_0 (min^{-1})	k_1 (min^{-1})	k_2 (min^{-1})	A_1	A_2
**Buffer**	0.6 +/- 0.1			0.94 +/- 0.02	
**Bulk coacervate phase**		1.0 x 10^{-2} +/- 0.1 x 10^{-2}	1.5 x 10^{-4} +/- 0.8 x 10^{-4}	0.23 +/- 0.03	0.77 +/- 0.03
**Coacervate microdroplets**		4.4 x 10^{-2} +/- 1.3x 10^{-2}	2.3 x 10^{-3} +/- 0.2 x 10^{-3}	0.31 +/- 0.20	0.69 +/- 0.20

Supplementary Table 3: Summary of observed rate constants from single exponential fit (k_0) and from biexponential fits (k_1, k_2)

	TAM-HH-mut (39-mer)	FAM-HH-subs (12-mer)	FAM-Cleaved substrate (6-mer)
CM-Dex / PLys	189 +/-14 s	22 +/- 3.5	4.2 +/- 1.4 s
PLys / ATP	4800 +/- 4300 s	800 +/- 400 s	19 +/- 7 s

Supplementary Table 4: Summary of the time constant (τ) of the fluorescence recovery of different length RNA from whole droplet FRAP of coacervate microdroplets prepared from a 4:1 molar ratio of either CM-Dex : PLys or PLys : ATP

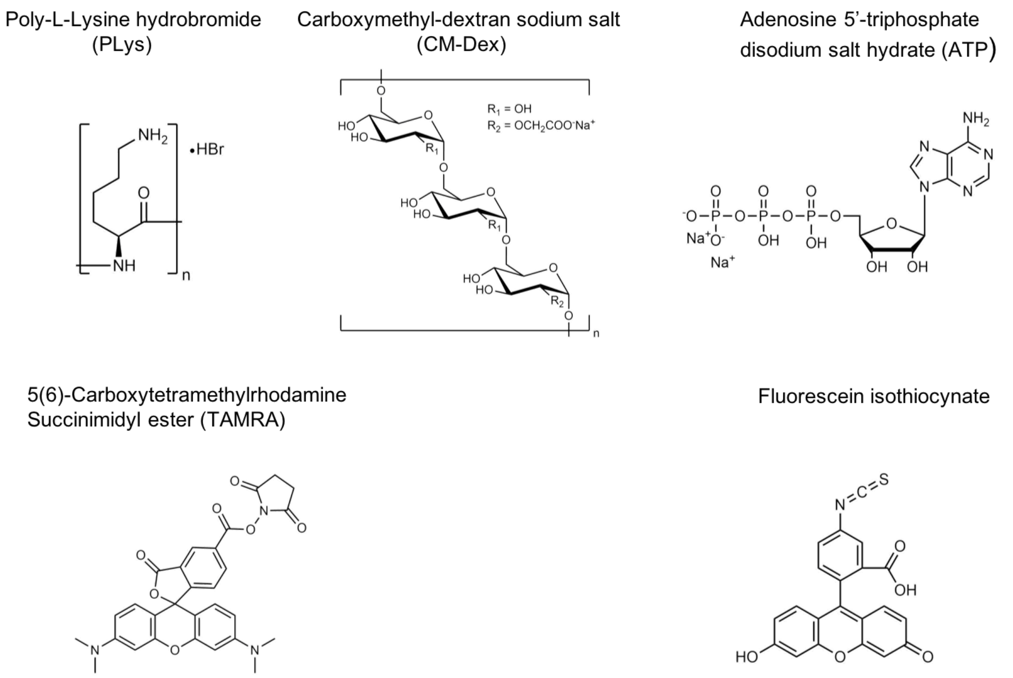

Supplementary Figure 1: Molecular structures of coacervate components Poly-L-Lysine (PLys), Carboxymethyl-dextran sodium salt (CM-Dex) and Adenosine 5' Triphosphate (ATP) and molecular structures of dye molecules, 5(6)-Carboxytetramethylrhodamine N-succinimidyl ester (TAMRA) and Fluorescein isothiocyanate isomer (FITC/FAM).

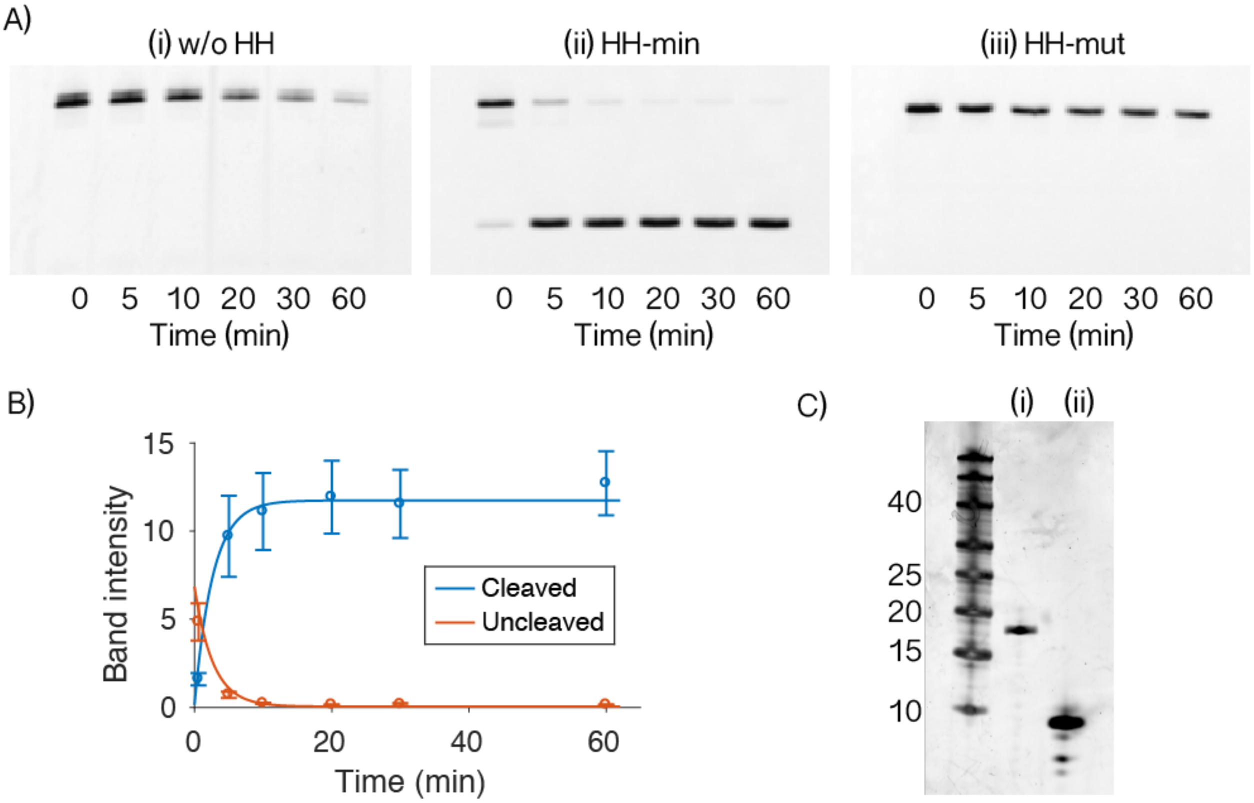

Supplementary Figure 2: **FRET assay for HH ribozyme in cleavage buffer** (10 mM Tris, 4 mM $MgCl_2$) at 25 ^oC. **(A)** Characterisation was undertaken by gel electrophoresis and fluorescence gel imaging without HH-min and 0.5 µM FRET-substrate (i), 1 µM HH-min and 0.5 µM FRET-substrate (ii) or 1 µM HH-mut and 0.5 µM FRET-substrate at 5 min, 10 min, 20 min, 30 min and 60 min. The gels show HH-min is active whilst HH-mut is inactive. **(B)** The kinetics of HH-min were determined by integrating the band intensities of the cleaved and uncleaved bands and performing a global fit to a single exponential (Equation 1,2), yielding a first order rate constant of 0.38 ± 0.05 min^{-1} (see materials and methods). Data points are an average of six independent measurements, error bars are standard deviation of the six measurements. Note that the in-gel fluorescent intensities of cleaved and uncleaved FAM-labelled RNA are different and are attributed to the FRET effect (Supplementary Figure 13B). **(C)** Denaturing PAGE gel electrophoresis (20% Acrylamide) of (i) FRET-substrate (12mer) and (ii) FAM- cleaved substrate (6 mer). Comparisons to the RNA molecular weight marker shows the FRET-substrate at 18 nucleotides and the FAM-cleaved substrate at 8 nucleotides. The increase in the molecular weight is attributed to the FAM and black hole quencher tags on the substrate and cleaved product respectively.

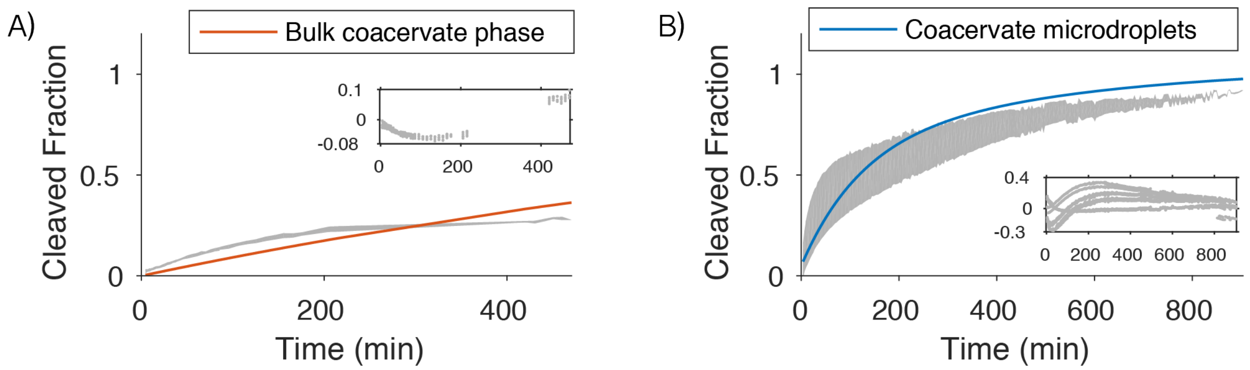

Supplementary Figure 3: Kinetic analysis for HH-min FRET assay in (A) bulk coacervate phase (CM-Dex: PLys, 4:1 final molar ratio) (A) or (B) a dispersion of coacervate microdroplets (CM-Dex:PLys, 4:1 final molar ratio). The assay was undertaken in limited substrate conditions at total concentrations of 1 µM for HH-min and 0.5 µM FRET-substrate. Time resolved data was either from **(A)** a plate reader experiments or **(B)** optical microscopy. The increase in fluorescence intensity from cleaved product (grey data) was plotted as a function of time and the thickness of the grey shaded region is the standard deviation from 5 repeats (A) and 12 individual droplets (B). Single exponential fits to the data show non-random residuals (inset) indicating that fits to single-exponential decays are not reliable.

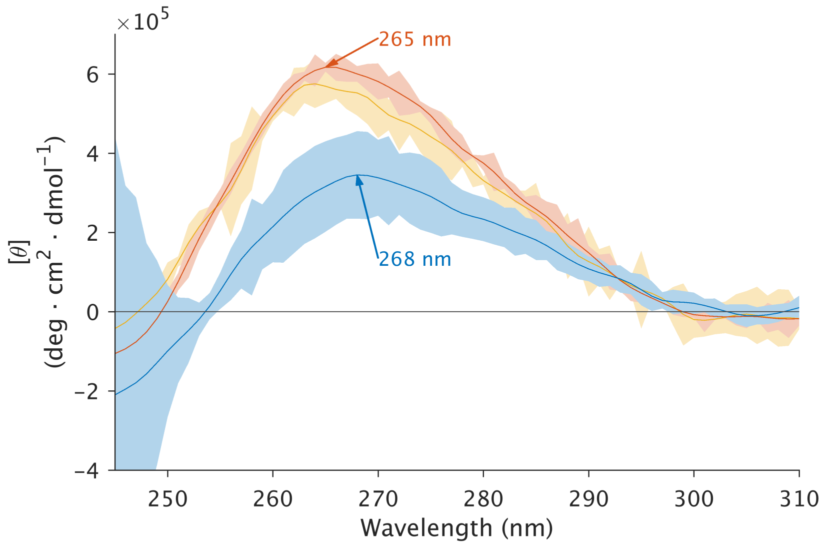

Supplementary Figure 4: Circular Dichroism Spectroscopy of 2 μM HH-mut in cleavage buffer (orange line), 1 μM HH-mut in cleavage buffer (yellow line) and 2 μM HH-mut in CM-Dex : PLys, 4:1 final molar ratio bulk coacervate phase (blue line) shown between 250 nm and 310 nm to focus on the RNA secondary structure [1]. The shaded region shows the standard deviation from 3 spectra, each an average of 5 repeats (orange and yellow) and 13 spectra, each an average of 10 repeats (blue). All spectra are shown with the background (either cleavage buffer or bulk polymer phase) removed.

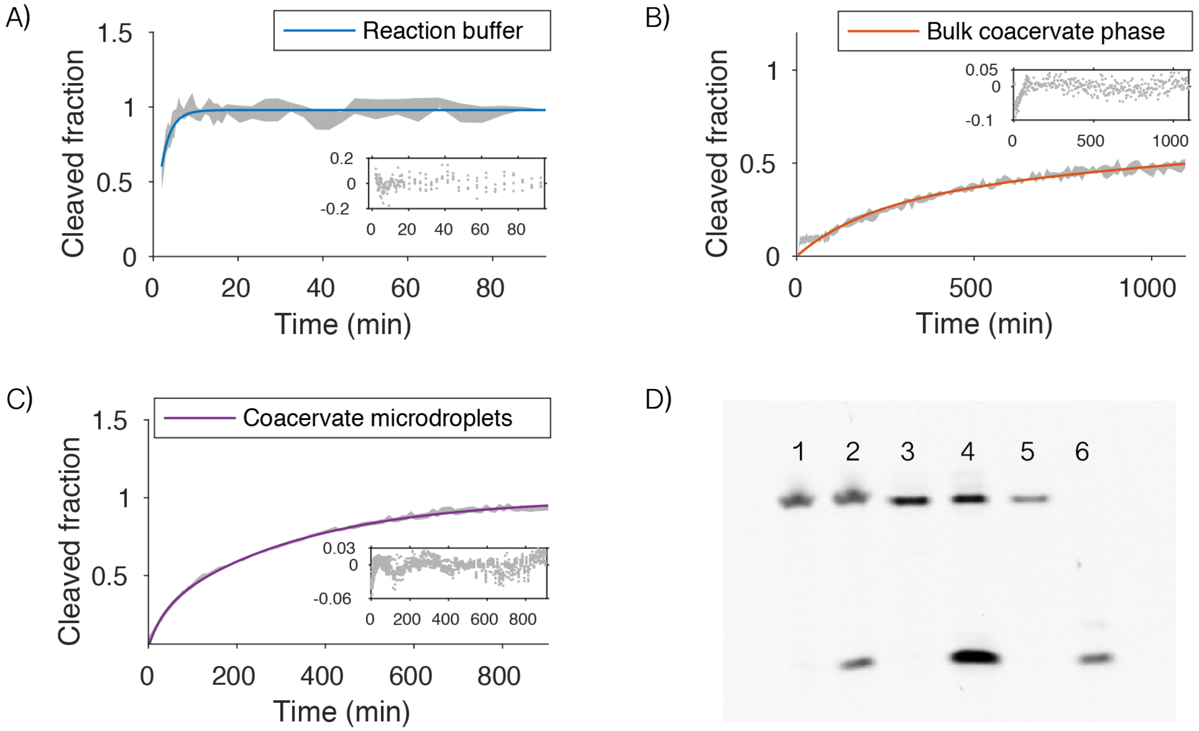

Supplementary Figure 5: Reproducibility of HH-min – FRET-substrate assay: The FRET assay was repeated with a different batch of HH-min and FRET-substrate (final concentrations of 1 µM for HH-min or HH-mut and 0.5 µM FRET-substrate) in cleavage buffer, bulk coacervate phase or coacervate microdroplets. Time-resolved kinetics was measured in **(A)** cleavage buffer (10 mM Tris•HCl pH 8 and 4 mM $MgCl_2$), or **(B)** bulk coacervate phase (CM-Dex : PLys, 4:1 final molar ratio), using a plate reader or **(C)** in microdroplets (CM-Dex : PLys, 4:1 final molar ratio) by fluorescence optical microscopy at final concentrations of 1 µM for HH-min and 0.5 µM FRET-substrate. Time traces of (A) could be fit with a single exponential (Equation 3) (k_0 = 0.5 +/- 0.1 min^{-1}, N=5). Time traces for (B) and (C) were fit with double exponentials (Equation 4) yielding rate constants and amplitudes of k_1 of 5 x 10^{-3} +/- 2 × 10^{-3} min^{-1} (A_1 = 0.31 +/- 0.05) and k_2 of 2.9 × 10^{-4} +/- 0.2 x 10^{-4} min^{-1} (A_2 = 0.69 +/- 0.05) for (B) N=3, and k_1 of 3.0 × 10^{-2} +/- 0.1 × 10^{-2} min^{-1} (A_1 = 0.24 +/- 0.04) and k_2 of 3.0 × 10^{-3} +/- 0.3 × 10^{-3} min^{-1} (A_2 = 0.76 +/- 0.03) for (C) N=11. The rate constants for the buffer and microdroplet are comparable to previous experiments (Supplementary Table 3) i.e. within the same order of magnitude. There are larger errors between repeats of the bulk coacervate phase which could be attributed to greater errors associated with sample handling of bulk coacervate phase. The difference in the rate constants between the coacervate microdroplets and the bulk coacervate phase is greater than the experimental error in the coacervate bulk phase. **(D)** Gel electrophoresis for determination of the fraction of cleaved cleaved product at specific time points obtained from substrate and cleavage band intensities for normalisation of kinetic curves after correcting for the FRET effect (1) HH-mut in bulk coacervate phase (0.01 cleavage after 270 min), (2) HH-min in bulk coacervate (0.26 cleavage after 270 min), (3) HH-mut in coacervate microdroplets (0.007 cleavage after 270 min), (4) HH-min in coacervate microdroplets (0.66 cleavage after 270 min), (5) HH-mut in cleavage buffer (0.005 cleavage after 90 min) (6) HH-min in reaction buffer (0.98 cleavage after 90 min). Shaded regions around the data represents the standard deviation.

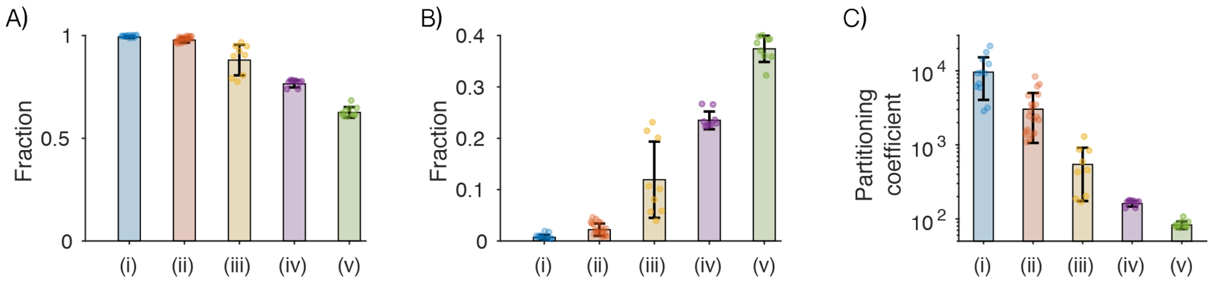

Supplementary Figure 6: Partitioning of three different length RNA (i) 39-mer TAM-HH-min, (ii) 12-mer FAM-substrate, (iii) 6-mer FAM-Cleaved-substrate and (iv) FITC (v) TAMRA in the 4:1 (final molar ratio) CM-Dex : PLys coacervate system (A-C). To obtain the partition coefficient 0.36 µM (total concentration) of either dye tagged RNA or dye, each sample was incubated in 150 µL CM-Dex / PLys coacervate microdroplet solution (3 µl bulk coacervate phase + 147 µl supernatant) and left to equilibrate for 10 min. The dispersion was then centrifuged and the supernatant separated from the coacervate phase. Either 3 µL of the bulk coacervate phase or 147 µl supernatant was made up to 150 µL by the addition of 147 µl RNA free supernatant or 3 µl RNA free coacervate, respectively. The fractions of dye tagged RNA or dye in the coacervate phase (**A**) and in the supernatant phase (**B**) were obtained spectroscopically. Partitioning coefficients were calculated using Equation 9-11 and plot for the CM-Dex / PLys coacervate system **(C)** with the y-axis plot on the logarithmic scale. The results show the general trend that longer length RNA partitions more into the coacervate phase compared to shorted length RNA with (i) 39-mer TAM-HH-min (K = 9600 +/- 5600, N=12), (ii) 12-mer FAM-substrate (K= 3000 +/- 2000, N=20), (iii) 6-mer FAM-Cleaved-substrate (K= 1500 +/- 300, N=9). The partition coefficients for (iv) FITC (K = 160+/-14, N=9) (v) TAMRA (K=83+/-10, N=9) are an order of magnitude less than the dye tagged RNA showing that partitioning is primarily driven by the nucleotide and not the dye molecule. Error bars are the standard deviations of repeat experiment.

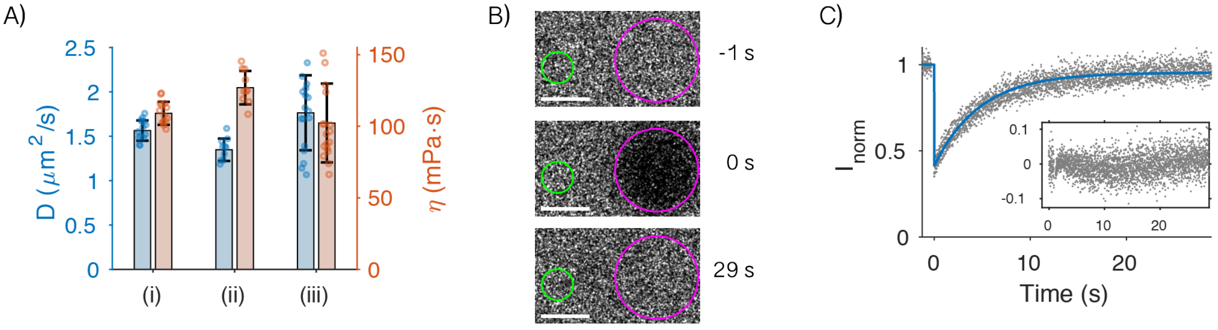

Supplementary Figure 7: FRAP of bulk coacervate phase (CM-Dex : PLys (4:1 final molar ratio)) and coacervate microdroplets (CM-Dex : PLys (4:1 molar ratio)) containing FAM-substrate **(A)** Diffusion coefficients (blue) (*D*) and viscosities (orange) (η) for (i) 0.36 µM FAM-substrate in bulk coacervate phase, D = 1.56 +/- 0.11 µm² / s, η = 109 +/- 8 mPa·s (N = 14), (ii) 24.3 μM FAM-substrate, which is the calculated concentration in microdroplets after accumulation based on the partitioning experiment (Supplementary Figure 6), in bulk coacervate phase, D = 1.35 +/- 0.13 µm² / s, η = 127 +/- 12 mPa·s (N = 10) and (iii) 24.3 µM FAM-substrate (overall concentration of 0.36 µM) within microdroplets D = 1.76 +/- 0.42 µm² / s, η = 102 +/- 28 mPa·s (N = 18), Error bars are standard deviations of repeated experiments. **(B)** Output frames of (ii) (scale bar 5 µm) from confocal imaging (63x) are shown at t = -1 s before bleaching, directly after bleaching (magenta circle, t = 0 s) and t = 29 s after bleaching. The fluorescence intensity was normalized against a reference (green circle). **(C)** Plots of normalised FRAP data for (ii) show the data distribution and averaged fit from fitting to Equation 5 (blue).

s

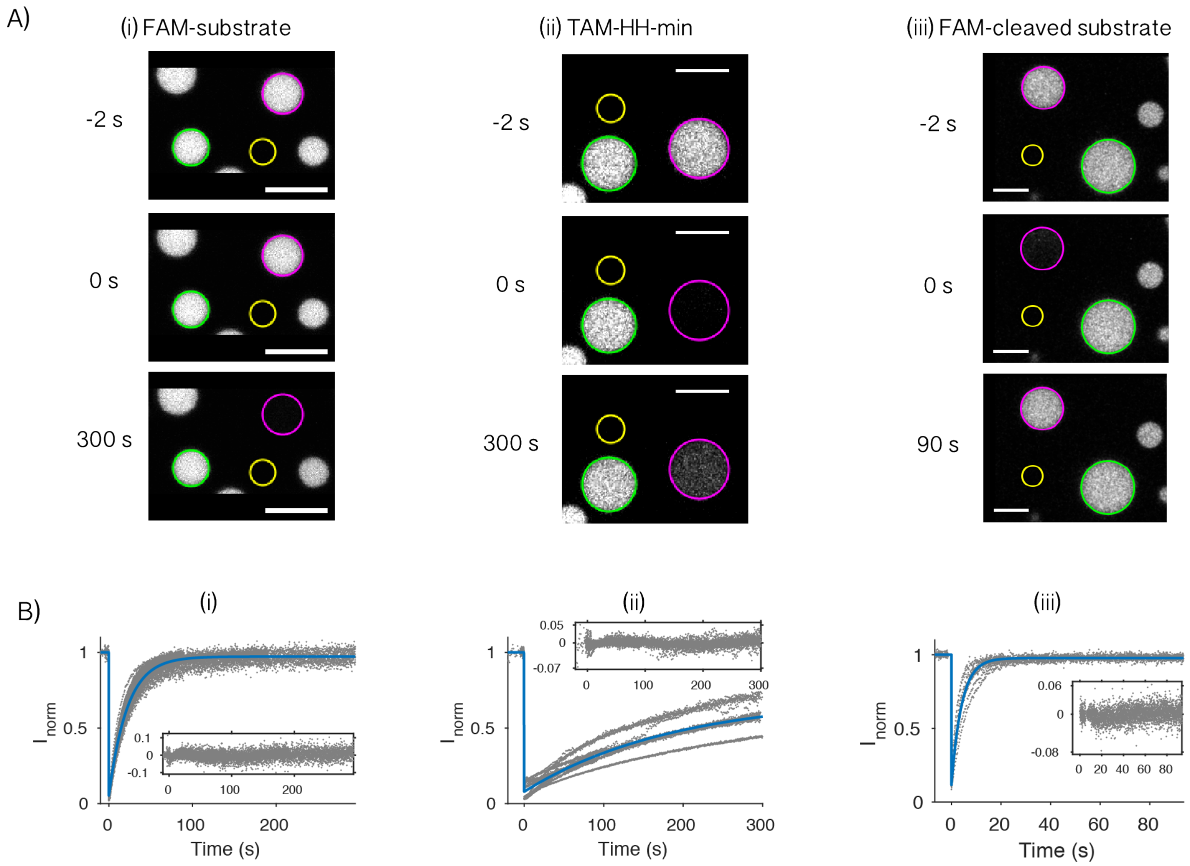

Supplementary Figure 8: **Whole droplet FRAP** for either 0.36 μM FAM-substrate (i, N = 20), 0.36 μM of TAM-HH-min (ii, N = 11) or 0.36 μM of FAM-cleaved substrate (iii, N = 10) in CM-Dex : Plys microdroplets (4:1 final molar ratio) undertaken using confocal microscopy with λ_{FAM} = 488 nm or λ_{TAM} = 514 nm and emission wavelengths λ_{FAM} = 479 to 665 (laser line blocking pin at 488 nm) nm or λ_{TAM} = 535 to 704 nm. Bleaching was undertaken with 355 and 405 nm lasers. **(A)** Fluorescence confocal microscopy images showing output frames at t = -2 s (before bleaching), t = 0 s (after bleaching) and either t = 300 s (i, ii) or t = 90 s (iii). The output images show complete recovery for FAM-substrate and FAM-cleaved substrate and incomplete recovery after 300 s for TAM-HH-min. Scale bars are 5 μm **(B)** Plots showing the fluorescence intensity of the bleached droplet (magenta) obtained as a function of time, plotted with the background removed (yellow) and corrected for photobleaching by normalisation with the reference (green) for FAM-substrate (i), TAM-HH-min (ii) and FAM-cleaved substrate (iii). The time constant τ (see Supplementary Table 4) was obtained by fitting data to Equation 5 (blue line is mean of fits). Analysis of the data shows only 70% recovery of TAM-HH-min after 300 s (ii).

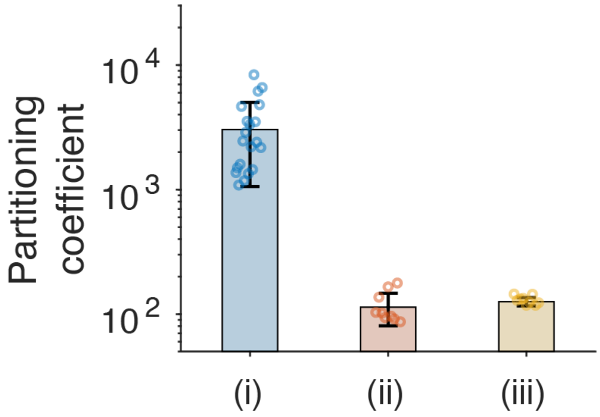

Supplementary Figure 9: Partitioning (K) of three different sequences RNA (i) 12-mer FAM-substrate (ii) 12-mer FAM-Flex (iii) 12-mer FAM-Tet. To obtain the partition coefficient 0.36 μM (total concentration) of FAM tagged RNA was incubated in 150 µl CM-Dex / PLys coacervate microdroplet solution (3 µl bulk coacervate phase + 147 µl supernatant) and left to equilibrate for 10 min. The dispersion was centrifuged and the supernatant phase separated from the coacervate phase. Either 3 µL of the bulk coacervate phase or 147 µl supernatant was made up to 150 µL by the addition of 147 µl RNA free supernatant or 3 µl RNA free coacervate, respectively. The fractions of FAM tagged RNA in the coacervate phase and in the supernatant phase were obtained spectroscopically and the partitioning coefficients were calculated using Equation 9-11 and plotted with the y-axis on the logarithmic scale. Corresponding partitioning coefficients are (i) 12-mer FAM-substrate (K= 3000 +/- 2000, N = 20), (ii) 12-mer FAM-Flex (K = 114 +/- 33, N = 9), (iii) 12-mer FAM-Tet (K= 126 +/- 10, N = 9). The results show that the partitioning of RNA is not only dependent on its length but also on its sequence/structure. Error bars represent the standard deviation from repeat experiments.

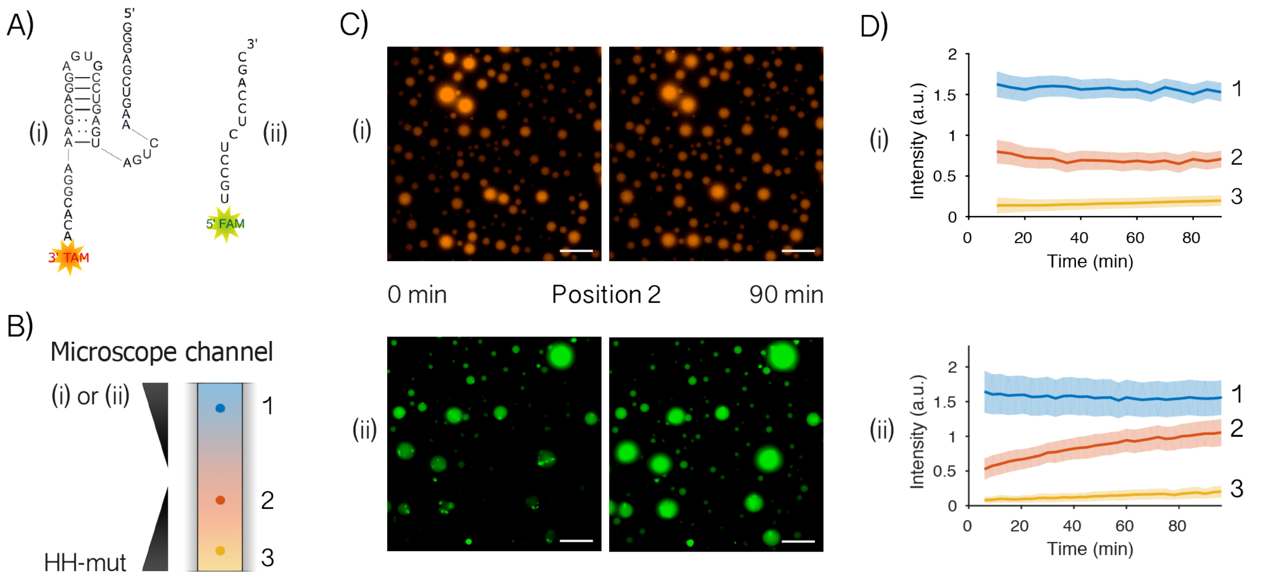

Supplementary Figure 10: Localisation experiments - Controls: **(A)** CM-Dex : PLys Coacervate microdroplets (4:1 final molar ratio) containing either 0.36 µM of TAM-HH-min (i) or FAM-substrate (ii) was loaded into a microscope channel and CM-Dex : PLys coacervate microdroplets (4:1 molar ratio) containing 0.36 µM HH-mut was loaded into the other end of the channel. Optical microscopy images were taken every 5 min for 90 min with λ_{exc} = 542 +/- 13.5 nm and λ_{em} = 593 +/- 23 nm and in the FAM channel with λ_{exc} = 469 +/- 20 nm and λ_{em} = 525.5 +/- 23.5 nm for TAM-HH-min and FAM-substrate respectively. **(B)** Optical microscopy images from position 3 shows no increase in fluorescence intensity after 90 min whilst optical microscopy images show an increase in fluorescence intensity of FAM-substrate in position 1 over time. **(C)** plots of the mean integrated fluorescence intensities (solid line) and the standard deviation (shaded area) of at least 7 droplets show no diffusion of TAM HH-min through the length of the channel (i) whilst the increase in fluorescence intensity in region 2 shows diffusion of the FAM-substrate from region 1.

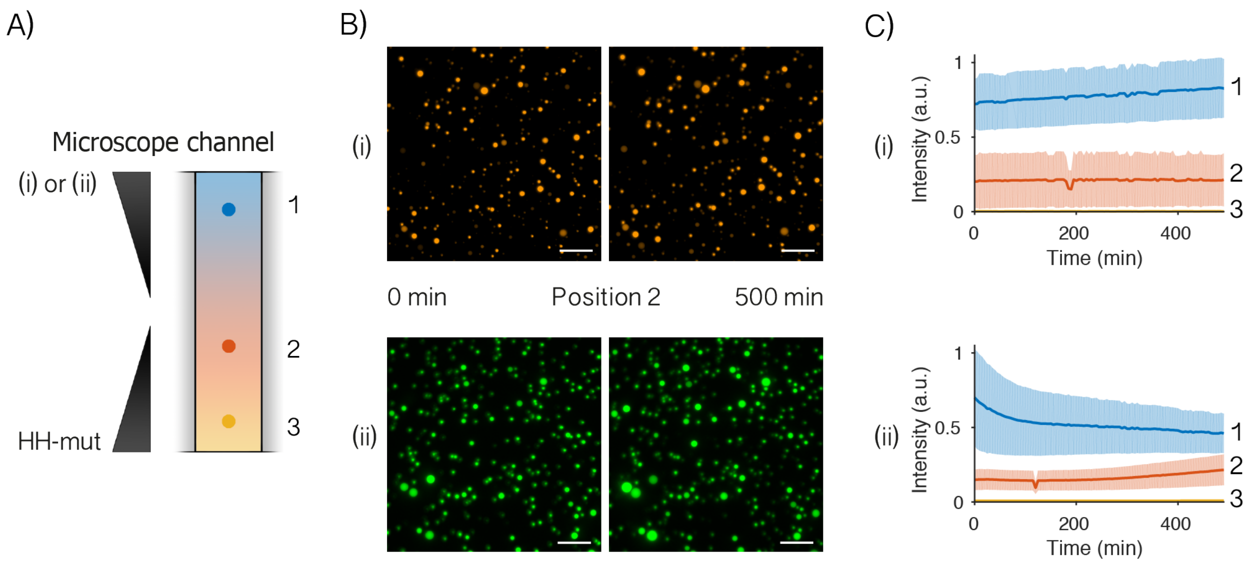

Supplementary Figure 11: **Localisation experiments for PLys : ATP (4:1 molar ratio) coacervate microdroplets.** **(A)** PLys : ATP Coacervate microdroplets containing either 0.4 μM of TAM-HH-min (i) or FAM-substrate (ii) were loaded into one end of a capillary channel and PLys : ATP coacervate microdroplets containing 0.4 μM HH-mut were loaded into the other end of the channel. Optical microscopy images were taken every 5 min for 500 min with λ_{exc} = 542 +/- 13.5 nm and λ_{em} = 593 +/- 23 nm (TAM-channel) or with λ_{exc} = 469 +/- 20 nm and λ_{em} = 525.5 +/- 23.5 nm (FAM-channel) for TAM-HH-min and FAM-substrate respectively. **(B)** Optical microscopy images from position 2 show no increase in fluorescence intensity after 500 min whilst optical microscopy images show a slight increase in fluorescence intensity of FAM-substrate in position 2 over time. **(C)** Plots of the mean integrated fluorescence intensities (solid line) and the standard deviation (shaded area) of at least 7 droplets as a function of time show no diffusion of TAM-HH-min through the length of the channel (i) whilst the increase in fluorescence intensity in region 2 shows diffusion of the FAM-substrate from region 1 to region 2 (ii) after 500 min.

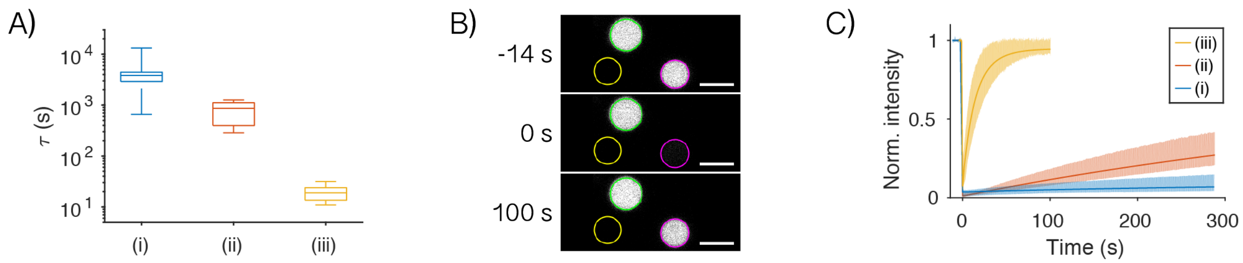

Supplementary Figure 12: Whole droplet FRAP of PLys: ATP (4:1 final molar ratio) microdroplet dispersions containing either 0.5 μM (total concentration) TAM-HH-min (39-mer) (blue) (i) or 0.5 μM (total concentration) FAM-substrate (12-mer) (red) (ii) or 0.5 μM (total concentration) FAM-cleaved substrate (6-mer) (yellow) (iii) using confocal microscopy with λ_{FAM} = 488 nm or λ_{TAM} = 514 nm and emission wavelengths λ_{FAM} = 479 to 665 nm (laser line blocking pin at 488 nm) or λ_{TAM} = 535 to 704 nm. Bleaching was undertaken with additional 355 and 405 nm lasers. **(A)** Box plot of the recovery half-time (τ) shows a general trend of increasing τ with longer length RNA indicative of less exchange for longer length RNA compared to shorter length RNA in PLys : ATP microdroplets; (i) 39-mer : τ = 4800 +/- 4300 s, N = 6; (ii) 12-mer : τ = 800 +/- 400 s, N=6; (iii) 6-mer : τ = 19 +/- 7 s, N=10 **(B)** Fluorescent Confocal microscopy images of whole droplet FRAP for 0.4 μM FAM-cleaved substrate showing output frames at t = -14 s (before bleaching), t = 0 s (at bleaching) and t = 100 s. The output images show full recovery of FAM-cleaved substrate. Scale bar: 5 μm. The fluorescence intensity as a function of time was obtained from the images from the bleached droplet (magenta), reference (green) and background (yellow). **(C)** Plot showing fluorescence recovery of the droplet after removal of the background and normalized to reference for TAM-HH-min (i) FAM- substrate (ii) and FAM-cleaved substrate (iii) the data was fit (solid line) to obtain τ. The large errors in τ for longer length RNA is attributed to fitting to a fluorescence recovery curves with incomplete recovery. The shaded region shows the data distribution (maximum to minima) from at least 6 droplets, as the exact time points for each measurement varies the standard deviation could not be determined in this instance. Analysis of the data shows full recovery for the shortest length (6 mer) RNA whilst the longer length RNA (12 mer and 39 mer) showed less than 40 % recovery after 290 s.

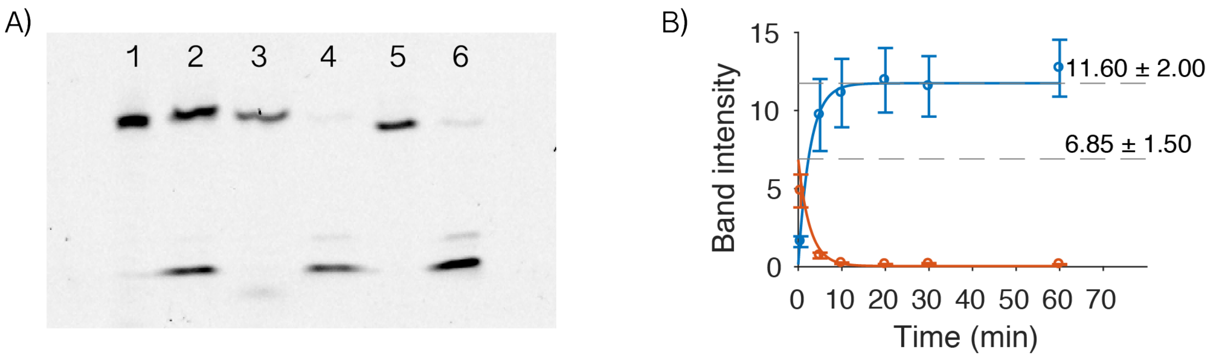

Supplementary Figure 13: Gel electrophoresis to obtain fraction of cleaved substrate from single turnover conditions for experiments reported in the main text Figures 1 and 3. **(A)** Gel electrophoresis of 1 μM HH-min or HH-mut and 0.5 μM of FRET-substrate in 4:1 final molar ratio CM-Dex : PLys in bulk coacervate phase or coacervate microdroplets). Lanes are (1) HH-mut in bulk coacervate phase (0.02 cleavage after 470 min), (2) HH-min in bulk coacervate (0.28 cleavage after 530 min), (3) HH-mut in coacervate microdroplets (0 cleavage), (4) HH-min in coacervate microdroplets (0.92 cleavage after 900 min), (5) HH-mut in cleavage buffer (0.02 cleavage after 45 min) (6) HH-min in cleavage buffer (0.94 cleavage after 45 min). **(B)** Extrapolation of maximal cleaved and uncleaved band intensities, at the start and end points of the reaction revealed a FRET effect of magnitude 1.69. Initial (6.85 +/- 1.50) and final intensities (11.60 +/- 2.00) are indicated with a dashed grey line (N=6), error bars represent the standard deviation of 6 experiments (see materials and methods). This was used to correct the cleaved and uncleaved band intensities.

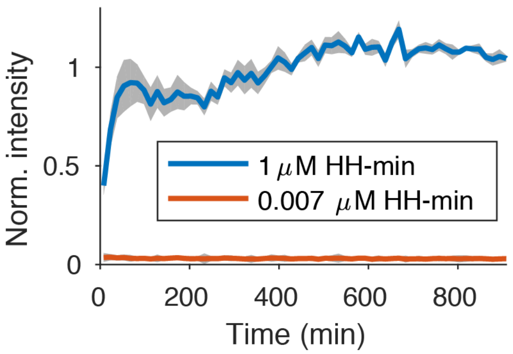

Supplementary Figure 14: FRET substrate cleavage in the aqueous phase from the microdroplet dispersion is negligible. As the partition coefficients show that there will be a small fraction of ribozyme within the surrounding aqueous phase of the coacervate droplets. Experiments were undertaken to determine the contribution of substrate cleavage in the aqueous phase to the overall measured kinetics within the coacervate microdroplets. The predicted concentration of HH-min within the supernatant phase was calculated from the partition coefficient 0.007 μM of HH-min and this was incubated under excess substrate conditions with 0.5 μM of FRET substrate within supernatant phase produced from CM-Dex:PLys (4:1 final molar ratio) coacervate microdroplet dispersions. The kinetics were obtained using a fluorescence plate reader (λ_{exc} = 485 nm and λ_{em} = 535 nm, 10 nm bandwidth). Comparisons to experiments containing 1 μM of HH-min and 0.5 μM of FRET substrate (blue data is the mean of 5 repeats and the grey shaded region the standard deviation of 5 repeats) show negligible amounts of cleaved product after 900 min.

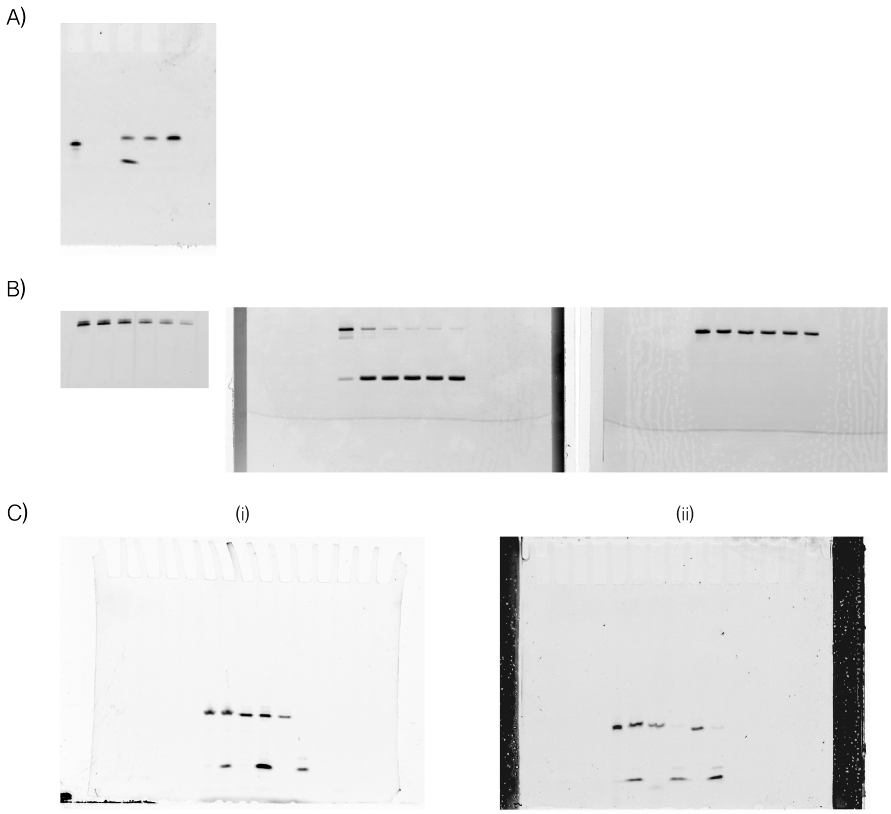

Supplementary Figure 15: Uncropped gels of gels shown in **(A)** Figure 1 **(B)** Supplementary Figure 2 (left- scan was taken of part of the gel) **(C)** (i) Supplementary Figure 5 (ii) Supplementary Figure 13

Supplementary References:

[1]Mikulecky, P. J., & Feig, A. L. (2002). Cold denaturation of the hammerhead ribozyme. Journal of the American Chemical Society, 124(6), 890-891.
